# Impact of a PACU bypass protocol on time from surgery completion to ward transfer and safety in patients undergoing spinal anesthesia: a retrospective cohort study

**DOI:** 10.1186/s40981-025-00831-7

**Published:** 2025-12-19

**Authors:** Emi Suda, Yukie Murooka, Tadanao Hiroki, Takashi Suto, Shigeru Saito

**Affiliations:** 1 Department of Anesthesiology, Gunma University Hospital, 3‑39‑22 Showa‑Machi, Maebashi, Gunma 371‑8511 Japan; 2 Department of Anesthesiology, Isesaki Municipal Hospital, 12-1 Tsunatori-honmachi, Isesaki, Gunma 372-0817 Japan

**Keywords:** Postanesthesia care unit bypass, Perioperative workflow, Operating room efficiency, Patient safety

## Abstract

**Introduction:**

Enhancing operating room (OR) efficiency and patient flow remains a persistent challenge in Japan. While post-anesthesia care units (PACU) are widely recognized as useful and have been routinely established in large hospitals, their implementation in small- and medium-sized hospitals remains limited owing to the costs of staffing and facility expansion. Safety concerns regarding direct ward transfer after surgery have also been raised. Therefore, intermediate PACU operational models feasible in smaller hospitals are needed. This study evaluated the effect of implementing a PACU bypass protocol on postoperative efficiency and patient safety.

**Methods:**

We retrospectively analyzed patients who underwent elective urological surgery under spinal anesthesia between June 2022 and October 2024. The PACU bypass protocol, implemented in 2023, enables eligible patients to be transferred directly from the OR to the ward when predefined criteria, including stable vital signs, anesthetic level at or below T4, and absence of postoperative symptoms, are met. The primary outcome was the duration from surgical completion to discharge from the PACU.

**Results:**

In total, 675 patients were included (214 in 2022, 225 in 2023, and 236 in 2024). The mean PACU stay will significantly decrease from 34 min in 2022 to 19 min in 2023 and 20 min in 2024 (*p* < 0.05). Two minor adverse events were reported in 2023, and none in 2024.

**Conclusions:**

Implementing the PACU bypass protocol significantly reduced the PACU stay without compromising patient safety, thereby enhancing perioperative efficiency. Continued protocol adherence and staff training are essential for sustained improvement.

## Background

Operating room (OR) efficiency is a critical component of perioperative management that balances workflow optimization and patient safety [1, 2]. As in many other healthcare systems in Japan, enhancing OR turnover, while maintaining high standards of care, has become a priority [3]. The post-anesthesia care unit (PACU) plays a key role in supporting postoperative safety, improving patient satisfaction, and contributing to efficient OR management. The necessity for PACUs has been widely recognized, and they have consequently been routinely established in many developed countries [4–6]. However, their implementation in Japan has been largely limited to large hospitals. According to a report in 2017, only 16.1% of institutions have an operational PACU, with the primarily limiting factor being the costs associated with securing adequate staffing and expanding facilities to maintain a unit with dedicated personnel, although these costs are often offset by surgical revenue [7]. At the same time, concerns have been raised regarding patient safety when patients are transferred directly to a ward after surgery. Therefore, there is a need to develop intermediate PACU operation models that are feasible in small- and medium-sized hospitals.

At the Isesaki Municipal Hospital, all patients undergoing either general or spinal anesthesia were routinely transferred to the PACU before returning to their wards. The same nurse provided care both intraoperatively and in the PACU to ensure the continuity of care and patient safety. While the PACU facilitates postoperative management, it also introduces delays in turnover time between surgeries. Although assigning dedicated PACU nurses—a common practice in many institutions—was considered impractical because of the extensive training required and the time-consuming nature of patient handoff communications. As an initial step towards reducing the interval between surgeries, we aimed to limit the number of patients requiring PACU care. In 2023, we implemented a structured ward-return protocol, referred to as the “PACU bypass protocol.” This protocol allows eligible patients undergoing spinal anesthesia to be discharged directly from the OR to the ward, bypassing the PACU, based on predefined physiological stability criteria. Two years after implementation, we conducted a retrospective cohort study to evaluate the impact of the protocol on the perioperative workflow efficiency, PACU stay duration, and patient safety.

## Methods

### Study design and setting

This retrospective cohort study was conducted at Isesaki Municipal Hospital, a secondary care facility in Gunma, Japan. This study included patients who underwent urological surgery under spinal anesthesia between June 26 and October 31 in 2022, 2023, and 2024. Spinal anesthesia is recognized as a safe and effective anesthetic technique for lower abdominal and urological procedures, with lower rates of postoperative complications than general anesthesia [8, 9]. This study was approved by the Institutional Review Board of the Isesaki Municipal Hospital (IRB No.2024-30).

### Participants

Eligible participants were adult patients who underwent elective urological surgery under spinal anesthesia. The exclusion criteria included conversion to general anesthesia or cases managed by non-staff anesthesiologists, because these factors could affect protocol adherence and data reliability. Follow-up was limited to the immediate postoperative period and concluded with either discharge from the PACU or direct transfer to the hospital ward.

### Variables

The primary outcome was the time from the end of the surgery to discharge from the PACU. The primary exposure variable was the year of surgery, categorized as 2022 (pre-protocol), 2023 (first year of implementation), or 2024 (second year of implementation). The potential confounders included age, sex, body weight, and surgical type. The secondary outcome was the operating room turnover time, defined as the interval from completion of anesthesia in the preceding patient to entry of the subsequent patient into the operating room. Cases were deemed eligible if urological surgeries were conducted on the same day, and both the preceding and subsequent procedures were conducted under spinal anesthesia. The exclusion criteria were overlapping operating room occupancy, emergency surgeries, cases managed by non-staff anesthesiologists, and cases where the patient did not enter the PACU.

### Bias data sources and measurement

Data were extracted from the anesthesia and electronic medical records. The primary outcome was calculated using the surgical end-time documented by the anesthesiologists and the PACU discharge time recorded by the nursing staff. The PACU discharge criteria, which had been in place prior to the implementation of the bypass protocol, were defined as stable vital signs without the need for special interventions; confirmation that the anesthetic level was at or below T4; and the absence of symptoms such as dyspnea, delirium, or shivering. Once these criteria were confirmed by the attending anesthesiologist, the responsible ward nurse was requested to arrange the patient’s transfer to the ward. Regarding the specific numerical thresholds, the same criteria as for PACU bypass were applied. With the introduction of the PACU bypass protocol, five additional predefined institutional criteria were established to determine eligibility: (1) At least 15 min had elapsed since the initiation of spinal anesthesia; (2) the final anesthetic level was at or below the fourth thoracic (T4) dermatome; (3) the patient’s mean noninvasive blood pressure remained within 30% of the baseline; (4) at least 5 mL/kg of intravenous fluid was administered; and (5) the patient exhibited no postoperative symptoms, including nausea or general discomfort. These criteria were evaluated by the attending anesthesiologist at the end of surgery and documented in the anesthesia records. The assessment methods and documentation procedures were consistent throughout the study. This simplified PACU bypass protocol, tailored specifically for spinal anesthesia, was developed based on previous reports [2, 10, 11] and adapted to fit our institution’s clinical context.

To minimize selection bias, consistent inclusion and exclusion criteria were applied throughout the study. Potential confounding factors were compared across groups; however, multivariate adjustment using regression analysis was not performed. Observer bias was reduced by using standardized documentation procedures.

### Quantitative variables

Continuous variables, including time to PACU discharge, were analyzed without categorization. When comparisons were made, non-parametric methods were used because of potentially skewed distributions.

### Statistical methods

Statistical analyses were performed using R version 4.3.1. The Kruskal–Wallis test with Bonferroni correction was used to compare continuous variables across the three groups. For pairwise comparisons, the Mann–Whitney U test was used. Categorical variables were analyzed using Fisher’s exact test. A *p*-value < 0.05 was considered statistically significant. No imputation was performed for missing data and no subgroup or sensitivity analyses were conducted.

### Ethical considerations

This study was approved by the Ethics Committee of the Isesaki Municipal Hospital (Committee No. 11000495, Approval No. 2024-30, approved on November 21, 2024). The requirement for informed consent was waived through an opt-out notification posted on the hospital website.

## Results

In total, 272, 301, and 279 patients underwent elective urological surgery under spinal anesthesia in 2022, 2023, and 2024, respectively. After applying the exclusion criteria, 214 patients in 2022, 225 in 2023, and 236 in 2024 were included in the final analysis (Table 1). The demographic characteristics, including age, sex, and type of surgery, did not differ significantly between the groups. The PACU bypass protocol was applied in 178 patients (79%) in 2023 and in 175 patients (74%) in 2024 (Table 2).

**Table 1:** Patient enrollment and final sample size following exclusions

		2022	2023	2024
Total cases		272	301	279
Excluded cases		58	76	43
	Conversion to general anesthesia	2	6	4
	Non-staff anesthesiologists	56	70	39
Analyzed cases		214	225	236

**Table 2:** Patient background and PACU bypass rule application rate

			2022		2023		2024		*p*-value
			*n* = 214		*n* = 225		*n* = 236		
Age			73	[66–78]	74	[66–79]	73	[66–78]	0.701
Sex	Male/Female (%)		181/33	(85/15)	186/39	(83/17)	189/47	(80/20)	0.457
Body weight	kg		61.4	[54.9–67.8]	62.1	[53.5–69.3]	62.2	[54.6–69.9]	0.874
Surgical procedure	n (%)	Prostate biopsy	83	(39)	81	(36)	94	(40)	
		TUR-Bt	39	(18)	37	(16)	37	(16)	
		TUL	23	(11)	26	(12)	30	(13)	
		HoLEP	10	(5)	5	(2)	14	(6)	
		Ureteral stent placement	12	(6)	11	(5)	13	(6)	
		Ureteral stent exchange	28	(13)	34	(15)	25	(11)	
		Brachytherapy	8	(4)	8	(4)	6	(3)	
		Others *1	11	(5)	23	(10)	17	(7)	
Rule application rate	n (%)			-	178	(79)	175	(74)	0.227

Median [IQR]

*1 TUR-Bt: Transurethral Resection of Bladder Tumor, TUL: Transurethral Lithotripsy, HoLEP: Holmium Laser Enucleation of the Prostate

The most common reasons for ineligibility included a spinal anesthesia duration of < 15 min, anesthetic level above the T4 dermatome, and episodes of intraoperative hypotension (Table 3).

**Table 3:** Rates and reasons for Non-Eligibility for the PACU bypass rule

	2023		2024	
	*n* = 47		*n* = 61	
Non-eligible cases, n (%)				
Spinal anesthesia duration < 15 min	12	(26)	22	(36)
Anesthetic level above the T4	7	(15)	7	(11)
Hypotension	8	(17)	5	(8)
Insufficient fluid infusion	0	(0)	1	(2)
Nausea or general discomfort	2	(4)	1	(2)
Others*1	18	(38)	25	(41)

*1 Desaturation/Oxygen supplementation, Shivering, Bradycardia, Use of sedatives, other than predefined exclusion criteria

The primary outcome, defined as the time from surgery completion to PACU discharge, had a mean value of 34 min in 2022. Following the implementation of the PACU bypass protocol, the average duration decreased to 19 and 20 min in 2023 and 2024, respectively (*p* < 0.05). This period also included the duration during which patients designated for PACU bypass remained in the PACU while awaiting transfer by the ward staff. Furthermore, intraoperative fluid administration significantly increased post-protocol implementation (*p* < 0.05) (Table 4). Regarding the secondary outcome, the operating room turnover times were recorded for 184, 189, and 173 cases in 2022, 2023, and 2024, respectively. After applying the exclusion criteria, 117, 103, and 116 patients were analyzed, respectively. The median turnover times were 28 min in 2022, 26 min in 2023, and 26 min in 2024, with no significant differences between the groups (*p* = 0.714).

**Table 4:** Comparison of outcomes across groups

		2022		2023		2024		*p*-value	2022 vs. 2023	2022 vs. 2024	2023 vs. 2024
		*n* = 214		*n* = 225		*n* = 236					
Time from surgery completion to PACU discharge *1	min	34	[28–40]	19	[14–25]	20	[16–29]	< 0.001	< 0.001	< 0.001	0.019
Surgical duration	min	12	[8–39.75]	15	[7–46]	11.5	[7–42.25]	0.895			
Anesthesia duration *2	min	24	[16–59]	25	[15–63]	24	[15–59.25]	0.842			
Operating room time *3	min	73	[57–102]	59	[41–93]	60.5	[44–99.5]	< 0.001	< 0.001	< 0.001	0.81
Infusion volume	mL	350	[250–500]	450	[300–600]	500	[330–650]	< 0.001	< 0.001	< 0.001	0.45
Infusion volume/body weight	mL/kg	5.7	[4.1–7.8]	7.3	[5.1–10.0]	7.7	[5.5–10.5]	< 0.001	< 0.001	< 0.001	0.58
Number of RRS activation events *4		0		2		0		-			
Operating room turnover cases		*n* = 117		*n* = 103		*n* = 116					
Operating room turnover time	min	28	[18–37]	26	[18–37]	25	[19–34.25.25]	0.714			

Median [IQR]

*1 This time period also encompassed the duration during which patients designated for PACU bypass remain in the PACU area while awaiting transfer by ward staff

*2 Anesthesia duration was defined as the period from the time of intrathecal drug administration to the end of surgery

*3 Operating room time was defined as the duration from entry into the operating room to discharge from the PACU*4 Statistical analysis was not performed owing to the very small number of events

Regarding patient safety, the number of rapid response system (RRS) activations was zero in 2022, two in 2023, and zero in 2024. In 2023, these two activities occurred because of minor complications of bradycardia and hypotension in the ward and were promptly resolved with atropine and fluid administration. Both cases were among patients eligible to undergo the PACU bypass protocol. No adverse events were reported by 2024, suggesting improved protocol adherence or patient safety.

## Discussion

Implementing the PACU bypass protocol for patients undergoing spinal anesthesia resulted in a significant reduction in the postoperative PACU stay without compromising patient safety. This reduction reflects improvements in perioperative workflow, contributing to enhanced OR turnover and more efficient resource utilization. Moreover, the reduction in the PACU stay likely contributed to the shorter operating room times, as the interval between surgery completion and patient transfer was minimized. Eligible patients no longer require routine observation in the PACU, thereby reducing transfer-related delays and enabling earlier release into the operating room, thus contributing to a more efficient workflow. In addition, the increased intraoperative fluid administration observed after protocol implementation may have improved the hemodynamic stability, further reducing the need for prolonged postoperative observation and supporting shortened operating room time.

Previous studies have indicated that early PACU discharge following spinal anesthesia can improve efficiency while maintaining safety. For example, Marino et al. reported that patients undergoing total joint arthroplasty were safely discharged from the PACU before full motor recovery, resulting in significantly shorter PACU stays and no increase in adverse events such as hypotension or nausea [12]. Our findings are consistent with these results and reinforce the value of structured bypass protocols in appropriately selected patients. However, no significant difference was observed in the operating room turnover time. Several factors may account for this, including an increase in the number of surgeries performed. Indeed, the total annual number of surgeries at our hospital increase from 4,555 in 2022 to 4,718 in 2023 and 4,822 in 2024. Consequently, it may be more difficult to prepare for subsequent surgeries in the available operating rooms to reduce turnover time. Moreover, the resources gained from the improved turnover efficiency may have been redirected to surgery in other departments. The absence of a significant difference, despite the growing surgical volume, suggests that the overall operating room efficiency was relatively well maintained.

The two minor adverse events (bradycardia and hypotension) observed in 2023 were promptly and effectively managed without sequelae. These events underscore the importance of careful patient selection, and intraoperative and postoperative monitoring. The absence of adverse events by 2024 suggests improved PACU bypass protocol adherence (including strict criteria for anesthesia duration, anesthetic efficacy range, and hypotension-induced exclusion), supporting the long-term safety and sustainability of this approach. Notably, the observed increase in intraoperative fluid volume following protocol implementation may have contributed to improved hemodynamic stability, thereby facilitating safer and more frequent direct ward transfers. Previous studies have demonstrated that appropriate fluid loading reduces the incidence of spinal anesthesia-induced hypotension, which is a common barrier to PACU discharge eligibility [13, 14]. Additionally, the effectiveness of our hospital’s RRS, implemented before the study period, played a crucial role in maintaining patient safety during direct ward transfers [15–17]. RRS activation criteria include: A change in respiratory rate to ≥ 35 breaths/min or ≤ 8 breaths/min; a newly observed decrease in peripheral capillary oxygen saturation (SpO₂) to ≤ 90% despite oxygen therapy; new-onset symptomatic tachycardia (≥ 130 bpm) or bradycardia (≤ 40 bpm); sustained tachycardia (≥ 160 bpm lasting >5 min); systolic blood pressure ≤ 80 mmHg, ≥ 200 mmHg, or a decrease of ≥ 40 mmHg from baseline; and newly developed oliguria (urine output ≤ 50 mL/4 hours). The adverse events observed in this study met activation criteria. The ability to detect and respond to early postoperative adverse events was largely supported by the effective functioning of the RRS, highlighting its essential role in sustaining the safe implementation of the PACU bypass protocol. In response to the 2023 adverse events and the corresponding RRS activations, the protocol was applied more rigorously in 2024, potentially contributing to a reduction in the number of RRS call cases. This more cautious approach likely reflects a heightened provider awareness of safety risks and underscores the need for ongoing staff training, protocol reinforcement, and quality assurance processes to maintain both safety and efficiency.

Our decision to include all cases regardless of the protocol eligibility was deliberate and intended to reflect the real-world clinical impact of the intervention. Restricting the analysis to protocol-adherent cases may have led to an overestimation of the benefits. By including ineligible patients, we were able to more accurately assess the practical effectiveness and limitations of the protocol in a typical clinical setting. This strategy may be particularly beneficial in secondary care hospitals where anesthesiology staff are often limited. By streamlining the patient flow without compromising safety, such protocols can help optimize care delivery in resource-constrained environments. This is especially relevant in aging societies such as Japan, where healthcare systems must increasingly balance quality and efficiency.

This study had several limitations. Firstly, it employed a retrospective design and was conducted at a single institution, which may limit the generalizability of the findings. In addition, the background of PACU use at our institution is unique. All patients were routinely observed in the PACU, and perioperative care in the PACU was provided by a nurse who was further responsible for intraoperative care. Therefore, the use of the PACU itself did not necessarily contribute to the improved operating room turnover, which may further limit the generalizability of our findings. Nevertheless, the consistency of the results across multiple years strengthens the reliability of our findings. Future multicenter prospective studies with larger sample sizes are warranted to further validate the efficacy and safety of this protocol. In addition, developing digital tools to support real-time eligibility assessment could enhance both accuracy and workflow integration in future implementations.

## Conclusions

Implementing the PACU bypass protocol for patients undergoing spinal anesthesia significantly reduced the postoperative PACU stay and improved perioperative workflow efficiency without compromising patient safety. The protocol yielded sustained reductions in recovery time over two consecutive years and demonstrated a favorable safety profile following initial implementation. These findings support the utility of protocol-based discharge criteria for enhancing OR efficiency, particularly in resource-constrained settings. Continued adherence to the protocol, combined with ongoing staff training and outcome monitoring, is essential to maintain these benefits and ensure safe long-term application.

## Data Availability

Data supporting the findings of this study are available from the corresponding author upon reasonable request.

## Abbreviations

PACU: Postanesthesia care unit
OR: Operating room
RRS: Rapid response system
SpO₂: Peripheral capillary oxygen saturation
